# Metabolic potential of the moderate halophile *Yangia* sp. ND199 for co‐production of polyhydroxyalkanoates and exopolysaccharides

**DOI:** 10.1002/mbo3.1160

**Published:** 2021-02-18

**Authors:** Luis Romero Soto, Habib Thabet, Reuben Maghembe, Denise Gameiro, Doan Van‐Thuoc, Tarek Dishisha, Rajni Hatti‐Kaul

**Affiliations:** ^1^ Division of Biotechnology Center for Chemistry and Chemical Engineering Lund University Lund Sweden; ^2^ Instituto de Investigación y Desarrollo de Procesos Químicos Universidad Mayor de San Andrés La Paz Bolivia; ^3^ Food Science and Technology Department Ibb University Ibb Yemen; ^4^ Department of Molecular Biology and Biotechnology College of Natural and Applied Sciences University of Dar es Salaam Dar es Salaam Tanzania; ^5^ Department of Biotechnology and Microbiology Faculty of Biology Hanoi National University of Education Hanoi Vietnam; ^6^ Department of Pharmaceutical Microbiology and Immunology Faculty of Pharmacy Beni‐Suef University Beni‐Suef Egypt

**Keywords:** co‐production, exopolysaccharide, moderate halophile, poly(3‐hydroxybutyrate), *Yangia* sp. ND199

## Abstract

*Yangia* sp. ND199 is a moderately halophilic bacterium isolated from mangrove samples in Northern Vietnam, which was earlier reported to grow on several sugars and glycerol to accumulate poly(hydroxyalkanoates) (PHA). In this study, the potential of the bacterium for co‐production of exopolysaccharides (EPS) and PHA was investigated. Genome sequence analysis of the closely related *Yangia* sp. CCB‐M3 isolated from mangroves in Malaysia revealed genes encoding enzymes participating in different EPS biosynthetic pathways. The effects of various cultivation parameters on the production of EPS and PHA were studied. The highest level of EPS (288 mg/L) was achieved using sucrose and yeast extract with 5% NaCl and 120 mM phosphate salts but with modest PHA accumulation (32% of cell dry weight, 1.3 g/L). Growth on fructose yielded the highest PHA concentration (85% of CDW, 3.3 g/L) at 90 mM phosphate and higher molecular weight EPS at 251 mg/L yield at 120 mM phosphate concentration. Analysis of EPS showed a predominance of glucose, followed by fructose and galactose, and minor amounts of acidic sugars.

## Graphical abstract

A moderate halophile Yangia sp. ND199, isolated from a mangrove sample in Northern Vietnam and known to accumulate polyhydroxyalkanoates (PHAs), was shown to have the potential for producing also exopolysaccharides (EPSs). Genome sequence analysis of the closely related Yangia sp. CCB‐M3 revealed the genes encoding enzymes participating in different EPS biosynthetic pathways. The effect of different cultivation parameters on the co‐production of PHA and EPS was studied; the EPS contained predominantly glucose followed by fructose and galactose, and minor amounts of acidic sugars.

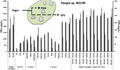

## 1 INTRODUCTION

Halophilic microorganisms from diverse thalassohaline and athalassohaline environments such as marine estuaries, solar salterns, and saline and soda lakes have acquired different genetic and physiological features that allow them to adapt to high salinity (Biswas & Paul, 2017). Extremely halophilic archaea, growing optimally at salt concentrations ranging between 15 and 30% (w/v), maintain an osmotic balance between the cell interior and the surrounding medium by accumulating a high concentration of inorganic ions in the cytoplasm. Moderate halophiles, growing at 3%‐15% (w/v) salt concentration, and halotolerant bacteria (with the ability to grow in the presence or absence of salt) accumulate high concentrations of compatible solutes (Oren, 2008; Roberts, 2005). Furthermore, the halophiles also produce pigments, biosurfactants, and extra‐ and intracellular polymers including exopolysaccharides (EPSs) and polyhydroxyalkanoates (PHAs), which apart from serving specific functions for microorganisms have interesting biotechnological applications (Biswas & Paul, 2017; Poli et al., 2011).

EPSs have various applications in the food, pharmaceutical, and other industries, and there is an increasing demand for the production of EPS from halophiles with better properties than those available (Castellane et al., 2014). PHAs are polyesters that have attracted interest for use as biodegradable plastics while possessing material properties of fossil‐based plastics (Sudesh et al., 2000). However, due to their higher production cost compared with fossil plastics, PHAs have not yet been able to compete on the market. While the feedstock and the polymer recovery from the cells constitute important cost‐determining factors, a co‐production with other chemicals/materials has been shown in earlier studies to have a favorable effect on the process economics (Guzmán et al., 2009; Li et al., 2017).

Several studies on the production of EPS and PHA individually by halophilic archaea and bacteria have been reported. Members of the haloarchaeal genera including *Haloferax*, *Haloarcula*, *Halococcus*, *Natronococcus,* and *Halobacterium* produce large amounts of EPS (Biswas & Paul, 2017). The same microbial groups, as well as *Haloterrigena*, *Natrialba*, *Natronobacterium*, *Haloquadratum,* and *Halorubrum* species, are also producers of PHA (Poli et al., 2011). *Haloferax mediterranei* is one of the best‐studied archaeal strains for the production of PHA (Alsafadi et al., 2020; Koller, 2017, 2019). Its attractive features are the ability to synthesize from unrelated carbon sources the copolymer poly(3‐hydroxybutyrate‐*co*‐3‐hydroxyvalerate) (P(3HB‐*co*‐3HV)) (Han et al., 2010)—with more suitable properties for the production of plastics than the homopolymer poly(3‐hydroxybutyrate) (PHB), the possibility of cultivating the organism under non‐sterile conditions, and the easy recovery of the polymer by lysing the cells just through the removal of salt (Rodriguez‐Valera & Lillo, 1992). *Hfx. mediterranei* is also the first halophile that was reported to produce EPS (Antón et al., 1988), and recent studies on co‐production of PHA and EPS by the organism have been reported (Cui et al., 2017a;b). Co‐production of these polymers was first reported in non‐halophilic microorganisms over two decades ago (Pal et al., 1999; Quagliano & Miyazaki, 1999; Tavernier et al., 1997).

Among the halophilic bacteria, the main EPS producers belong to the genera *Halomonas*, *Alteromonas,* and *Idiomarina* (Biswas & Paul, 2017; Sahana & Rekha, 2019). Members of the genus *Halomonas*, the most prevalent moderately halophilic bacteria isolated from different locations, are the most common producers of EPS with diverse physicochemical properties of interest in industrial and environmental applications (Biswas & Paul, 2017). Most of the EPS from halophilic bacteria has been characterized as sulfated heteropolysaccharides, with a significant amount of uronic acid and interesting features such as jellifying, emulsifying, metal binding, or biosurfactant properties (Arias et al., 2003; Biswas et al., 2015; Gutierrez et al., 2020; Martinez‐Checa et al., 2002; Mata et al., 2006). One of the exceptions so far seems to be *Halomonas smyrnensis* producing levan, a long linear homopolysaccharide of fructose residues (Ates & Oner, 2017; Tohme et al., 2018). The *Halomonas* species are also dominant among halophilic PHA‐producing bacteria (Koller, 2017; Quillaguamán et al., 2005, 2007, 2008); the possibility of polymer production by *Halomonas* TD01 under unsterile conditions was also demonstrated (Tan et al., 2011).

*Yangia* is a genus belonging to the *Roseobacter* group within the family Rhodobacteraceae, members of which have been widely found in marine environments. *Yangia pacifica* DX5‐10, isolated from coastal sediment of the East China Sea in the western Pacific Ocean, has earlier been reported to accumulate PHB (Dai et al., 2006). Subsequently, *Yangia* sp. ND199, isolated from mangroves in the Nam Dinh province in northern Vietnam, was shown to produce the copolymer P(3HB‐*co*‐3HV) from structurally unrelated carbon sources (Phong et al., 2016; Van‐Thuoc et al., 2012, 2015), a characteristic similar to *Hfx. mediterranei* (Han et al., 2010), but uncommon among bacterial species. A later study reported a P(3HB‐*co*‐3HV) producing *Yangia* sp. CCB‐MM3 isolated from mangrove soil samples from the west coast of Peninsular Malaysia, and its genome sequence, that revealed a pathway for the production of the copolymer (Lau et al., 2017). Phylogenetic analyses have shown the three isolates to be very closely related; *Yangia* sp. ND199 and CCB‐MM3 having 98.4% and 98.8% 16S rRNA gene sequence identity, respectively, with the type strain DX5‐10 (Lau et al., 2017; Van‐Thuoc et al., 2012). This study demonstrates the ability of *Yangia* sp. ND199 to produce EPS and PHA, and the effect of different environmental parameters on the production of these polymers. The metabolic capacity of the organism to produce EPS was further analyzed using genome analysis of the closely related *Yangia* sp. CCB‐MM3.

## 2 MATERIALS AND METHODS

### 2.1 Chemicals and culture medium components

Yeast extract was procured from Duchefa Biochemie (Haarlem, the Netherlands), peptone was procured from Merck (NJ, USA), while granulated agar, monosodium glutamate, standard PHB, standard PHBV, and polymer molecular weight standards were obtained from Sigma‐Aldrich (MO, USA). All other chemicals of analytical grade were obtained from VWR (Stockholm, Sweden).

### 2.2 Microorganisms, culture conditions, and preculture preparation

*Yangia sp*. ND199 (available at Vietnam Type Culture Collection (http://vccm.vast.vn) under accession number VCCM 14081), previously isolated from soil samples collected in mangrove forests in Nam Dinh province, Northern Vietnam (Van‐Thuoc et al., 2012), was propagated on modified solid HM medium (HM‐1) containing per liter: 50 g NaCl, 0.25 g MgSO_4_·7H_2_O, 0.09 g CaCl_2_·2H_2_O, 0.5 g KCl, 0.06 g NaBr, 5 g peptone, 10 g yeast extract, 1 g glucose, and 20 g granulated agar (pH 7.0) (Quillaguamán et al., 2004). A single colony of the bacterial culture, grown for 18 h at 32 °C, was transferred to 50 mL of HM‐1 medium in 250 Erlenmeyer flasks, and the culture was incubated for 12 h at 32 °C and 180 rpm. Subsequently, 1 mL aliquot of the culture was used to inoculate 50 mL of HM‐1 medium that was incubated under similar conditions for 8 h and then used as the inoculum for shake flask experiments.

### 2.3 Shake flask cultivation for simultaneous production of PHA and EPS

Aliquots of the freshly prepared inoculum (2.5 mL) were used to inoculate 50 mL of HM‐3 medium containing per liter (unless otherwise stated) 25 g NaCl, 0.25 g NH_4_Cl, 0.25 g MgSO_4_·7H_2_O, 0.09 g CaCl_2_·2H_2_O, 0.5 g KCl, 0.06 g NaBr, 2.5 g K_2_HPO_4_, 1.5 g KH_2_PO_4_, 2 g yeast extract, and 20 g glucose (pH 7). All the medium components were autoclaved together except glucose, magnesium sulfate, and phosphate buffer salts, which were sterilized separately and added to the medium before inoculation. The cultures were incubated at 32 °C and 180 rpm for 48 h, and samples were collected at different intervals for monitoring cell density, pH, and concentration of the carbon source. The PHA and EPS concentrations were determined once after 48 h based on previous reports on *Yangia* sp. ND199 (Phong et al., 2016).

The co‐production of PHA and EPS was also evaluated in the presence of 5% (w/v) NaCl and higher concentrations of phosphate salts.

To study the effect of different carbon sources on co‐production of PHA and EPS, glucose was replaced by 20 g/L of a monosaccharide (fructose, galactose, arabinose), a disaccharide (sucrose, lactose, maltose), or a polyol (glycerol).

The effect of various nitrogen sources on PHA and EPS co‐production was investigated using 2 g/L of ammonium chloride, ammonium sulfate, ammonium nitrate, and monosodium glutamate, instead of yeast extract in the HM‐3 medium.

The additional batches that combined the higher phosphate buffer concentrations with 5% NaCl in the medium were used to identify the best conditions for EPS and PHA production.

### 2.4 EPS recovery

The culture broth (35 mL) was centrifuged at 3000 **×**g for 15 min at 4 °C, the cell pellet was discarded, and the cell‐free supernatant was used for the recovery of free EPS as described in earlier works (Leroy & De Vuyst, 2016; Sardari et al., 2017). Briefly, the supernatant was mixed with 3 times its volume of 99.5% ethanol and stored overnight at 4 °C in bottles to precipitate the EPS. Subsequently, the mixture was centrifuged at 12 500 **×**g, 4 °C for 25 min, and the supernatant was discarded. The precipitate was transferred to 50‐mL Falcon tubes, frozen, and freeze‐dried for 24 h. To remove proteins, the powder was dissolved in 10% (w/v) TCA solution (3 mL) and then centrifuged at 2400 **×**g and 4°C for 15 min. The pellet was discarded, and the supernatant was frozen and freeze‐dried immediately to yield a white powder.

To determine the EPS concentration, the powder was weighed and the concentration in milligrams per liter of cultivation broth was calculated.

### 2.5 Bioinformatic analysis of EPS biosynthetic genes in Yangia sp. CCB‐MM3 genome

The *Yangia* sp. CCB‐MM3 genome was retrieved from the NCBI database (https://www.ncbi.nlm.nih.gov/genome/45565?genome_assembly_id=279568), and EPS biosynthetic genes were individually searched through KEGG. The enzymes identified in KEGG were then reannotated via BLAST in the UniProt KB database. The operons for the individual genes were then searched for through the BioCyc database to gain insight into the possible gene cluster based on orthologs from other bacteria.

### 2.6 Analytical procedures

#### 2.6.1 Cell growth

Cell growth was monitored by measuring OD at 620 nm using an Ultrospec 1000 spectrophotometer (Pharmacia Biotech, Uppsala, Sweden) and correlating it with cell dry weight (CDW). To determine CDW, 10 mL of the culture samples was centrifuged in preweighed Falcon tubes at 4000 x g for 15 min, the pellet was washed once with 10 mL distilled water, and then centrifuged and lyophilized until a constant weight was reached. The centrifuge tube was weighed again; a decrease in weight with respect to the original weight yielded the CDW.

#### 2.6.2 Substrate consumption

The concentrations of carbon sources were determined using a Jasco HPLC equipped with Jasco Intelligent autosampler (Tokyo, Japan). Five microliters of the filtered, properly diluted sample (in 50 mM potassium phosphate buffer, pH 7) was injected in an Aminex HPX‐87P chromatographic column (Bio‐Rad, Richmond, CA, USA) that was placed in a chromatographic oven (Shimadzu, Japan) at 65 °C and connected to a guard de‐ashing column. The compounds were separated using Millipore quality water flowing at a rate of 0.4 mL/min, and the detection was done using an RI detector (ERC, Kawaguchi, Japan).

#### 2.6.3 Determination of PHA concentration

The PHA content was analyzed using gas chromatography according to the method described earlier (Huijberts et al., 1994). Approximately 10 mg of freeze‐dried cells was mixed with 1 mL chloroform, to which was added 1 mL of methanol solution (containing 15% (v/v) sulfuric acid and 0.4% (w/v) benzoic acid) before incubating the mixture at 100 °C for 3 h to convert the PHA monomers formed to their methyl esters. Subsequently, the mixture was cooled to room temperature, and 0.5 mL of distilled water was added and then shaken for 30 s. The chloroform layer was transferred to another tube and 2 µL sample volume was injected into the gas chromatography column (Varian, Factor Four Capillary Column, Varian, 15 m x 0.25 mm), with injection temperature set at 250 °C and detector temperature at 240 °C. The column temperature for the first 5 min was maintained at 60 °C and then increased at a rate of 3 °C/min to 120 °C. Standard PHB was used for calibration. All of the analysis was performed in triplicate. PHA content (wt% of cell dry weight) of the cells and PHA concentration (per liter culture broth, g/L) were determined as reported previously (Quillaguamán et al., 2007).

#### 2.6.4 Determination of EPS molecular weight

EPS samples were analyzed using the AF4 field‐flow fractionation technique (Fuentes et al., 2018) using an Eclipse 3+ system (Wyatt Technology, Dernbach, Germany) connected to a Dawn Heleos II (MALS) detector (Wyatt Technology, Dernbach, Germany) and an Optilab T‐rEX differential refractive index detector (Wyatt Technology, Dernbach, Germany), both operating at a wavelength of 658 nm. An Agilent 1100 series isocratic pump (Agilent Technologies, Waldbronn, Germany) with an in‐line vacuum degasser and an Agilent 1100 series autosampler delivered the carrier flow and handled the sample injection into the AF4 separation channel. A polyvinylidene fluoride membrane with a 100 nm pore size (Millipore, Bedford, MA, USA) was placed between the pump and channel to ensure that particle‐free carrier entered the channel. β‐Glucan standards (35 to 650 kDa) were used as molecular weight standards, in a concentration range of 0.01–0.1 mg/mL. The standards and EPS samples were delivered into the system in a liquid carrier containing 0.02% (w/v) NaN_3_ and 10 mM NaNO_3_ in Milli‐Q water.

#### 2.6.5 Total carbohydrate analysis of EPS

The total carbohydrate analysis of EPS was conducted using a colorimetric method as described previously (Dubois et al., 1956). Briefly, 1 mL of a solution containing 10 mg of powdered sample was first diluted 100‐fold to achieve a concentration within the standard curve (concentration 10–100 mg/L). Two hundred microliters of the diluted sample were mixed with 200 µL of phenol solution in a glass tube, and then 1 mL of H_2_SO_4_ was rapidly added against the liquid surface. The tubes were allowed to stand for 10 min and shaken before placing them in a water bath at 25–30°C for 10–20 min followed by measuring absorbance in a spectrophotometer at 490 nm. Millipore quality water (200 µL) was used as a blank. The EPS concentration in the samples was determined using the standard curve made with glucose, considering the dilution factor.

#### 2.6.6 Monosaccharide analysis of EPS

Monosaccharides present in the EPS were determined by acid hydrolysis of the polymer followed by chromatographic analysis (Saeman et al., 1963). Ten‐milligram portions of the freeze‐dried EPS samples were weighed into tubes and 175 μL of 72% (w/w) sulfuric acid was added, followed by incubation for 60 min in a water bath at 30 °C. Subsequently, the sulfuric acid concentration was reduced to 4% (w/w) and the samples were heated to 100 °C for 3 h. Finally, the tubes were cooled, vortexed, and centrifuged for 1 min at 9400 x g at room temperature, the supernatant (1 mL) was transferred to a small tube, and pH was set to 5 with 0.1 M Ba(OH)_2_. Samples were then centrifuged for 5 min at 2700 × g and filtered through a 0.2‐μm membrane filter. The released monomeric sugars were separated on HPAEC‐PAD (ICS‐5000; Dionex, Sunnyvale, CA) using a CarboPac PA20 Column (150 mm × 3 mm, 6.5 µm) fitted with a guard column (30 mm × 3 mm) and mobile phase comprising 0.75 mM NaOH (Merck, Solna, Sweden) at a flow rate of 0.5 mL/min. Standards of glucose, galactose, and fructose were used for calibration of the equipment.

#### 2.6.7 FTIR characterization of EPS

Fourier transform infrared (FTIR) spectroscopy was used for the identification of the functional groups in the *Yangia* sp. ND199 EPS. Infrared spectra of the purified EPS samples were recorded in the 4000–400 cm^−1^ region using an FTIR system (Nicolet iS 5; Thermo Fisher Scientific, USA).

#### 2.6.8 Statistical analysis

All experiments were done in two independent replicates, and the results are the average of these replicates ±SD. The significance was determined using Student’s *t* test.

## 3 RESULTS

### 3.1 Genome sequence analysis of the related Yangia sp. CCB‐MM3 for EPS biosynthetic genes

**FIGURE A1:**
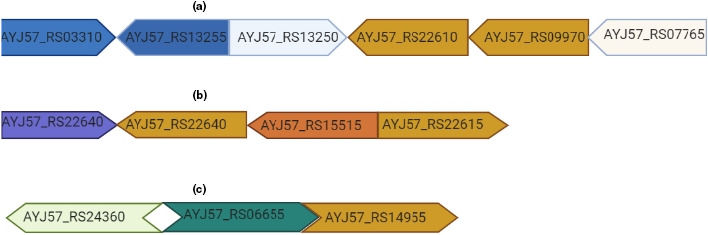
Virtual EPS biosynthetic gene clusters for A: Wxz/Wzy pathway, B: ABC transporter pathway, and C: Synthase pathway

Owing to the close similarity of the known *Yangia* spp., we analyzed the available genome sequence of *Yangia* sp. CCB‐MM3 to identify any genes related to EPS synthesis, which occurs via different pathways in bacteria: the Wzx/Wzy‐dependent pathway, the ATP‐binding cassette (ABC) transporter‐dependent pathway, the synthase‐dependent pathway, and the extracellular synthesis catalyzed by a single sucrase protein (Ates, 2015). A detailed analysis of the genome annotation report indicated that *Yangia* sp. CCB‐MM3 contains several genes related to virtually all EPS biosynthetic pathways (Table A1). Also, the KEGG search showed that the metabolic pathway map for the halophile does not directly highlight EPS biosynthetic gene clusters and the genes do not comprise a significant operon. Hence, we reannotated the proteins identified as EPS biosynthetic gene candidates based on the UniProt KB database and selected the most relevant functions associated with their role in the established EPS biosynthetic pathways (Ates, 2015; Islam & Lam, 2013), also integrating their orthologs from other species. The same genes have separate operons in the BioCyc database, with a more elaborate organization. Interestingly, functional annotation of the proteins based on UniProt convincingly suggests that the genes could play a role in EPS biosynthesis, although from separate operon systems. A virtual cluster for each biosynthetic pathway is, hence, proposed by focusing on the coding sequence and function of each gene, ignoring its BioCyc operon (Fig. A1).

### 3.2 Influence of environmental factors on PHA and EPS production by Yangia sp. ND199

#### 3.2.1 Effect of NaCl concentration

**FIGURE A2:**
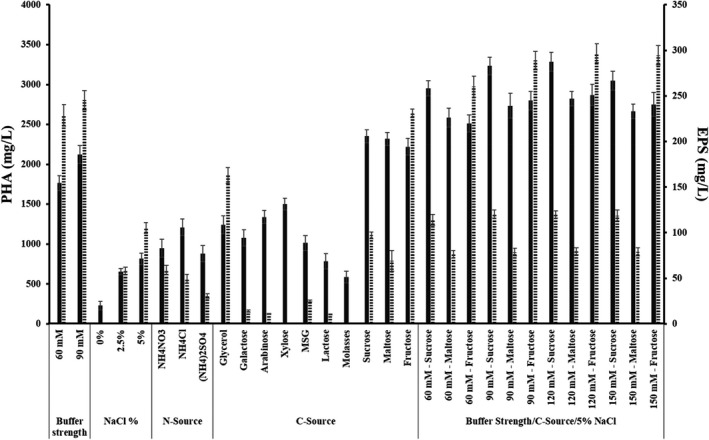
Production of EPS (■) and PHA (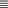) by *Yangia* sp. ND199 after 48 hours under different culture conditions

*Yangia* ND199 was grown in the HM‐I medium containing 0%‐5% (w/v) NaCl. As expected, negligible cell growth and no PHA accumulation were detected in the medium without NaCl; however, approximately 20 mg/L of EPS was present in the culture supernatant when analyzed after cultivation for 48 h. Increases in salt concentration to 2.5% and 5% (w/v) resulted in increases in EPS concentration to 57.1 ± 3.6 and 92 ± 6.4 mg/L, respectively, and significant increases in the EPS molecular weight (Table 1, Figure A2).

**TABLE 1:** Effect of different medium components on EPS and PHA production by *Yangia* sp. ND199. Unless specified, the carbon source used is glucose, the nitrogen source is yeast extract, and the concentration of phosphate salts in the medium (1x) is equivalent to 30 mM.

Sample	Culture conditions	CDW (g/L)	EPS (mg/L)	EPS MW (kDa)	PHA (mg/L)	PHA content (% CDW)
*Buffer concentration, glucose 20 g/L, 2.5% NaCl*						
0	1x Buffer	3.12 ± 0.19	57.1 ± 3.6	16.6 ± 0.8	664.3 ± 43.4	21.2 ± 1.4
1	2x Buffer	3.57 ± 0.21	68.4 ± 4.1	18.2 ± 1.1	1217.4 ± 62.7	34.1 ± 2.1
2	3x Buffer	3.92 ± 0.24	82.3 ± 4.4	20.1 ± 1.8	1391.6 ± 70.6	35.5 ± 2.8
*NaCl concentration, medium with 20 g/L glucose*						
3	No NaCl	0.31 ± 0.03	20 ± 4.6	4.1 ± 0.3	0	0
4	2.5% NaCl	3.12 ± 0.19	57.1 ± 3.6	16.6 ± 0.8	664.3 ± 43.4	21.2 ± 1.4
5	5% NaCl	3.78 ± 0.18	92 ± 6.4	189.5 ± 9.5	1200.8 ± 67.5	31.7 ± 1.8
*Nitrogen source (2 g/L), 20 g/L glucose, 5% NaCl*						
6	NH_4_NO_3_	1.98 ± 0.15	82.9 ± 10.1	7.7 ± 0.3	675.4 ± 60.2	34.1 ± 3.1
7	NH_4_Cl	2.07 ± 0.15	105.7 ± 9.1	6.4 ± 0.3	562.1 ± 57.8	27.1 ± 1.7
8	(NH_4_)_2_SO_4_	1.49 ± 0.09	77.1 ± 8.9	3.7 ± 0.1	348.9 ± 26.5	23.4 ± 1.8
9	MSG	2.48 ± 0.16	88.6 ± 9.6	4.3 ± 0.2	287.6 ± 9.6	11.6 ± 0.9
*Carbon source (20 g/L) with 5% NaCl*						
10	Glycerol	3.74 ± 0.22	108.6 ± 10.1	3.6 ± 0.2	1859.7 ± 96.4	49.7 ± 4.4
11	Galactose	1.18 ± 0.07	94.3 ± 8.9	4.5 ± 0.3	158.4 ± 14.5	13.4 ± 1.4
12	Arabinose	1.24 ± 0.09	117.1 ± 7.2	4.9 ± 0.4	122.7 ± 4.8	9.8 ± 0.7
13	Xylose	1.67 ± 0.11	131.4 ± 6.3	7.8 ± 0.4	0	0
14	Lactose	0.76 ± 0.06	68.6 ± 8.4	4.8 ± 0.3	117.4 ± 5.4	15.4 ± 0.9
15	Molasses	0.66 ± 0.05	51.4 ± 6.5	3.9 ± 0.1	0	0
16	Maltose	3.15 ± 0.19	202.9 ± 7.2	126.2 ± 6.3	788.3 ± 50.6	25 ± 1.8
17	Fructose	3.46 ± 0.16	194.3 ± 9.3	**266.8** ± **14.3**	**2638.5 ± 75.4**	**76.3 ± 6.8**
18	Sucrose	3.63 ± 0.25	**205.7** ± **7.4**	227.6 ± 13.1	1109.6 ± 43.8	30.6 ± 2.3
*Buffer concentration/C source in medium with 5% NaCl*						
19	2x Buffer /sucrose	3.92 ± 0.29	258.3 ± 8.4	214.4 ± 10.1	1303.3 ± 67.5	33.2 ± 2.5
20	3x Buffer /sucrose	4.13 ± 0.28	282.8 ± 9.5	218.5 ± 11.1	1378.7 ± 50.6	33.4 ± 1.3
21	4x Buffer/sucrose	4.27 ± 0.33	**287.6 ± 10.3**	239.9 ± 10.7	1370.4 ± 45.8	32.1 ± 1.1
22	5x Buffer /sucrose	4.35 ± 0.34	266.7 ± 10.3	227.5 ± 10.9	1356.8 ± 72.3	31.1 ± 1.7
23	2x Buffer /maltose	3.51 ± 0.20	226.1 ± 10.3	142.1 ± 7.8	868.6 ± 48.2	24.7 ± 1.9
24	3x Buffer /maltose	3.67 ± 0.22	239.2 ± 13.5	155.2 ± 6.3	899.4 ± 48.2	24.4 ± 1.2
25	4x Buffer/maltose	3.88 ± 0.26	246.9 ± 8.0	168.4 ± 8.1	907.9 ± 45.7	23.3 ± 1.2
26	5x Buffer /maltose	3.98 ± 0.25	233.1 ± 8.1	151.2 ± 6.1	900.5 ± 63.7	22.6 ± 1.3
27	2x Buffer/fructose	3.81 ± 0.24	219.8 ± 9.5	**259.6** ± **12.2**	2974.4 ± 132.5	77.8 ± 4.1
28	3x Buffer/fructose	3.89 ± 0.23	244.9 ± 10.1	247.1 ± 12.4	3301.6 ± 145.7	**84.7 ± 7.7**
29	4x Buffer/fructose	4.14 ± 0.29	251.1 ± 11.6	255.7 ± 13.3	**3392.2 ± 160.5**	81.8 ± 6.3
30	5x Buffer/fructose	4.19 ± 0.32	240.7 ± 13.1	249.6 ± 11.8	3367.2 ± 150.8	79.8 ± 5.4

The values in bold are the among the highest values obtained.

#### 3.2.2 Effect of the buffering system

Cultivation in shake flasks with glucose as the carbon source, 2.5% NaCl, and initial pH of 7 gave a cell dry weight of 2.44 ± 0.14 g/L in the initial 24 h (growth rate of 0.102 g/L.h), followed by a significant reduction in growth rate (0.009 g/L.h), increasing just 0.21 g CDW/L over the subsequent 24 h. The decrease in growth was closely associated with increased acidity of the medium, and the pH dropping to 6.5 and 5.0 at 24 and 36 h of cultivation, respectively. The HM‐I medium contained phosphate salts at low concentration (30 mM) as buffering agents, which may not provide sufficient capacity for pH control. The effect of increasing the concentration of phosphate salts (up to fivefold) in the medium on cell growth, PHA, and EPS formation by *Yangia* sp. ND199 was, thus, studied with different sugars as carbon sources (Table 1). Around a 20% increase in cell density was observed with a fivefold increase in phosphate levels to 150 mM. The PHA content, as well as EPS concentration, increased (up to 29%) until a fourfold increase in phosphate concentration to 120 mM, while the culture was maintained above 6 during the entire cultivation period (Table 1).

#### 3.2.3 Effect of nitrogen source

The cell growth, PHA, and EPS production were all influenced by the nitrogen source used. Yeast extract was the optimum nitrogen source for cell growth and PHA production and replacing it with ammonium salts or monosodium glutamate resulted in a drastic reduction in PHA concentration (Table 1). Slightly higher levels of EPS were obtained using NH_4_Cl as the nitrogen source; however, the polysaccharide was of low molecular weight (in the range of 3.7–7.7 kDa compared with 189.5 kDa with yeast extract) (Table 1).

#### 3.2.4 Effect of the carbon source

Concerning the C sources used for cultivation of *Yangia* sp. ND199, the EPS concentration was the highest (287.6 ± 10.3 g/L) with sucrose, followed by fructose and then maltose. Despite this, EPS molecular weight was highest (267 kDa) when fructose was used at the lowest phosphate buffer concentration (30 mM) and showed a downward trend in the order fructose, sucrose, and maltose (Table 1, Figure A2). EPS with relatively low molecular weight (126–168 kDa) was obtained using maltose as a C source, while other sugars (arabinose, lactose, galactose) led to a much lower molecular weight of the polysaccharide (3–8 kDa).

**FIGURE 1:**
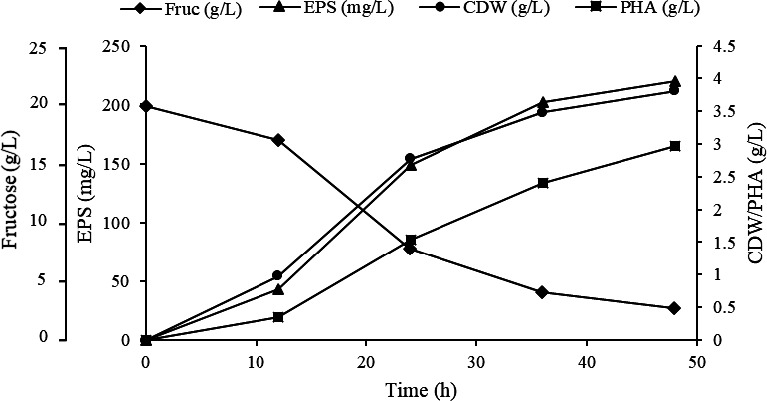
Growth profile of *Yangia* sp. ND199 cultured on fructose, with 60 mM phosphate salts concentration for 48 hours. Symbols: (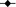) fructose, (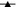) EPS, (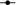) cell dry weight, and (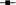) PHA.

Production of both EPS and PHA by *Yangia* sp. ND199 was seen to be growth‐associated (Figure 1). Their concentrations varied between 219.8–287.6 mg/L EPS and 0.87–3.39 g/L PHA after 48 h of cultivation in media with different carbon sources, yeast extract as a nitrogen source, and 5% (w/v) NaCl. The highest cell growth (4.3 ± g/L) and EPS production (287.6 ± 10.3 mg/L) were obtained using sucrose as the carbon source, 5% (w/v) NaCl, and 120 mM phosphate salts and at initial pH of 7; the PHA content was, however, much lower (32% w/w CDW). Fructose was the best carbon source for PHA production (85% w/w CDW) at 5% (w/v) NaCl and 90 mM phosphate salts and was also close to sucrose with regard to cell growth (4.1 ± 0.29 g/L) and EPS production (251 ± 11.6 mg/L) of a higher molecular weight at phosphate salt concentration of 120 mM (Table 1).

### 3.3 EPS composition

Analysis of EPS composition produced by *Yangia* sp. ND199 when grown using sucrose as the C source and at 5% w/v NaCl concentration revealed glucose to be the predominant sugar, ranging from 55.9 to 61.1% of total sugars (Table 2). Fructose also made up a substantial fraction of the polymer, while galactose was present in small amounts. Two additional peaks adjacent to each other with a longer retention time than fructose were observed in the chromatogram, suggesting the presence of sugar acids (glucuronic and galacturonic acids). Increasing the buffer concentration led to a relative increase in the glucose content, while both the fructose and galactose contents were slightly decreased.

**TABLE 2:** Monomeric content of the EPS produced by *Yangia* sp. ND199 in a medium with 5% (w/v) NaCl and sucrose as carbon source and different concentrations of phosphate buffer.

Sample No. (from Table 1)	Buffer concentration^a^	Composition (% of total)^b^		
		Glucose	Fructose	Galactose
20	3× buffer	55.9	32.7	7.8
21	4× buffer	58.3	30.2	7.4
22	5× buffer	61.1	28.7	6.3

^a^ The normal buffer concentration (1x) in the medium is 30 mM.

^b^ The EPS also contains a minor proportion of acidic sugars.

**FIGURE 2:**
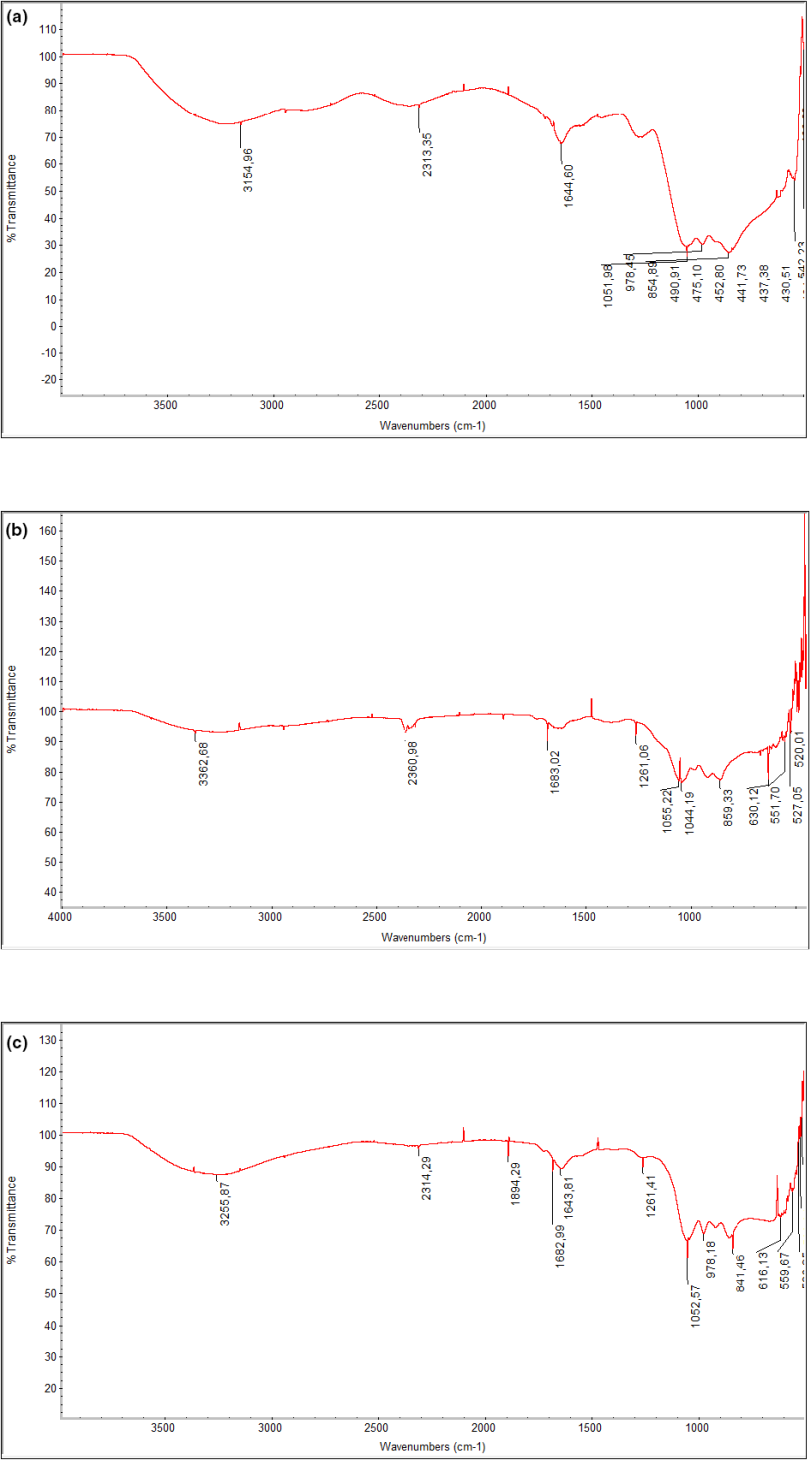
Fourier transform infrared spectra of EPS samples obtained from the medium with (a) fructose, (b) sucrose, and (c) glucose as a carbon source for *Yangia* sp. ND199.

FTIR analysis of EPS samples obtained from the ND199 cultures grown under different conditions was performed. Absorption bands common to all EPS samples were in the range of 3100–3400 cm^‐1^, characteristic of the polysaccharide (Gu et al., 2017; Sardari et al., 2017). The other important absorption bands observed in several samples were at 2360 cm^−1^, 1644 cm^−1^, 1219 cm^−1^, 1051 cm^−1^, 978 cm^−1^, 840–860 cm^−1^, and 630 cm^−1^ (Figure 2).

## 4 DISCUSSION

Halophilic microorganisms constitute a promising source of industrially important polymers PHA and EPS. Among the moderate halophiles, most reports so far have dealt with *Halomonas* species. This study describes the relatively less known moderately halophilic *Yangia* species; the three reported strains isolated from the East China Sea, Malaysia, and Northern Vietnam, are all similar, and produce PHA, including the copolymer PHBV (Dai et al., 2006; Lau et al., 2017; Phong et al., 2016; van‐Thuoc et al., 2012, 2015).

The *Yangia* sp. CCB‐MM3 genome has been sequenced, and its analysis has confirmed the presence of a complete methylmalonyl‐CoA pathway for the production of propionyl‐CoA and gene cluster for P(3HB‐*co*‐3HV) production (Lau et al., 2017). Besides possessing two PHA synthase genes, CCB‐MM3 also possesses two PHA depolymerase genes. The 16S rRNA gene sequence comparison showed that *Yangia* sp. ND199 has 98.43% identity with the type strain *Y. pacifica* DX5‐10 and *Yangia* sp. CCB‐MM3 (Lau et al., 2017; Van‐Thuoc et al., 2012). Due to such close similarity, we analyzed the genome sequence of *Yangia* sp. CCB‐MM3 and located genes related to EPS biosynthesis. Although not closely located, the genes‐utilizing different operon systems are likely to influence EPS biosynthesis. The production of EPS was further demonstrated experimentally, and the effect of different cultivation parameters on cell growth, EPS, and PHA production in shake flasks was investigated.

We have previously shown the versatility of *Yangia* sp. ND199 to accept several C6 sugars and glycerol as carbon sources and produce both PHB and P(3HB‐*co*‐3HV) (with HV content of 1.3%−7.4%), except for fructose that yielded only the homopolymer PHB (Van‐Thuoc et al., 2015). The halophile requires at least 2.5% NaCl for cell growth, and 4.5%‐5% (w/v) NaCl for optimum growth and PHA production (Van‐Thuoc et al., 2015). The halophiles are more prone to cell lysis in the absence of salt, which would provide a facile means for PHA extraction after its production in a saline medium. In this study, increasing the salt concentration to 5% (w/v) was even found to be favorable for the production of EPS. The salt concentration was not increased further since the earlier studies indicated a decrease in both cell growth and PHA levels at a higher salt concentration (Phong et al., 2016; Van‐Thuoc et al., 2015).

While most moderate halophiles have a broad pH range of growth between 5 and 10 (Ventosa et al., 1998), *Yangia* sp. ND199 was reported to exhibit the highest growth rate at pH 6.7 when grown with glycerol and yeast extract in a bioreactor and increasing the pH to 7.7 significantly lowered the growth rate and PHA accumulation (Van‐Thuoc et al., 2015). The reduction in the cell growth rate with time during the cultivation of ND199 was accompanied by increasing acidity of the medium and could be prevented by increasing the concentration of the buffer salts in the medium, which also had a favorable effect on the biosynthesis of both biopolymers. As shown in Table 1, the highest cell mass was obtained with a fivefold increase in phosphate salt, whereas the highest PHA and EPS levels were achieved with only a fourfold increase in phosphate. While phosphate limitation is often associated with increased PHA and EPS formation by several bacteria, there are also reports showing the opposite behavior (Concórdio‐Reis et al., 2018; Melanie et al., 2018). In the case of *Yangia* cultivation in shake flasks, the stimulation of cell growth by an increase in phosphate levels could be ascribed to better pH control and for meeting the nutritional requirements of the bacteria, which in turn also has a positive effect on PHA and EPS production to a certain extent. The specific effect of phosphate levels on the synthesis of polymers by *Yangia* sp. ND199 would need to be studied under controlled conditions in a bioreactor.

According to the results obtained in this study, sucrose was the best carbon source for EPS synthesis followed by fructose, however considering the co‐production of EPS and PHA, fructose is a superior carbon source for *Yangia* sp. ND199. Furthermore, yeast extract was a more suitable nitrogen source than inorganic nitrogen sources for cell growth and production of both polymers (although slightly higher EPS levels were obtained using NH_4_Cl as the N source) as also shown recently for PHA production in *Hfx. mediterranei* (Alsafadi et al., 2020). The carbon/nitrogen (C/N) ratio for the *Yangia* cultivation was 30–35 with yeast extract, and 10–16 with the other N‐sources depending on the carbon source used. The obtained results are in agreement with those reported earlier by Cui et al. (2017a), who observed that the highest accumulation of PHA polymer by *Hfx. mediterranei* occurs under N‐limiting conditions (C/N ratio of 35), while the highest EPS production occurs in an excess of nitrogen (C/N ratio of 5) with NH_4_Cl as an N source (Cui et al., 2017a).

The monosaccharide analysis of the *Yangia* sp. ND199 EPS showed a predominance of glucose followed by fructose and galactose, which make up approximately 96% of the total sugars (Table 2), while acidic sugars (with carboxylic groups) comprise a minor proportion of the polymer. While glucose and galactose seem to be commonly found in EPS, the presence of fructose in heteropolymers is less frequent and has been reported in EPS from *Hfx. prahovense* (Enache et al., 2012) and the EPS comprising the homopolymer levan produced by *H. smyrnensis* (Ates et al., 2017; Tohme et al., 2018). Characterization of EPS using FTIR revealed absorption band characteristic of sugars and polysaccharides (Figure 2). The absorption bands in the range of 3100–3400 cm^‐1^ result from the O‐H stretching vibration of the polysaccharide and are characteristic of the carbohydrate ring and confer water solubility of the polysaccharide. The absence of bands in the region of 2600 and 2500 cm^−1^ indicates that EPS does not contain sulfhydryl groups (Gu et al., 2017). The absorption band at 2360 cm^−1^ could be assigned to the C‐H stretching of methyl or methylene groups, usually present in hexoses such as glucose or galactose (Ismail & Nampoothiri, 2010). No absorption peaks around the region of 1700–1770 cm^−1^ attributed to C=O stretching of carbonyls in esters were observed (Kielak et al., 2017). The peak in the region of 1644 cm^−1^ would represent the absorption band of carboxylic groups, suggesting the presence of acidic sugars (Deshmukh et al., 2017; Kielak et al., 2017), and similarly, the peak at 1219 cm^−1^ observed in some samples could be attributed to C‐O stretching in ether or alcohol groups (Gu et al., 2017; Kanamarlapudi & Muddada, 2017). The peak at 1051 cm^−1^ indicates a possible α (1→6) glucosidic bond with certain flexibility (Gu et al., 2017), while the absorption band at 978 cm^−1^ suggests the vibrations of the glycosidic bonds C‐O‐C and the peak in the region of 840–860 cm^‐1^ are characteristic of α‐D‐glucan (Kanamarlapudi & Muddada, 2017). The band at 630 cm^‐1^ is due to the out‐of‐plane bending of –OH groups (Deshmukh et al., 2017).

The molecular weight of the *Yangia* EPS is within the high molecular weight range produced by halophilic and non‐halophilic microorganisms, some of which like xanthan are used as thickening agents in the food industry (Freitas et al., 2011; Imeson, 2009). EPS with molecular weights greater than 200 kDa could also be obtained using sucrose and glucose as carbon sources.

This study adds *Yangia* species to the group of halophiles that have the metabolic potential for the production of PHA and EPS. The organism is attractive because of its previously reported ability to produce copolymer P(3HB‐*co*‐3HV). While carbon and nitrogen sources and the concentrations of salts had significant effects on the production of polymers in *Yangia*, the amount of EPS produced so far (60–70 mg/g CDW) was low compared with that reported for other halophiles such as *Halomonas alkaliantarctica, Halomonas eurihalina*, *Halomonas maura, Halomonas xianhensis*, *H. smyrnensis,* and *Hfx. mediterranei* (Biswas & Paul, 2017a,b; Deshmukh et al., 2017; Sarilmiser et al., 2015; Cui et al., 2017b), but in the same range as that for *Halomonas ventosae* (Mata et al., 2006). Further studies are needed to determine the conditions for increasing the production of EPS, its characteristics, and its potential applications. A primary requirement for increasing the production of polymers is to increase cell growth as well as to trigger the synthesis of polymers. Hence, cultivation parameters such as optimal C/N ratio, aeration, phosphate concentration, and pH would need to be investigated under controlled conditions. Also, applying different modes of cultivation, such as fed‐batch and two‐stage cultivation in which the first stage is designed for optimal cell growth and the second for optimal production of the polymers, would have a significant impact on the production. It would further be interesting to express the metabolic pathways for PHA and EPS in a heterologous host for enabling their characterization and providing means for increasing their production under defined conditions.

## 5 ETHICS STATEMENT

None required.

## CONFLICT OF INTEREST

None declared.

## AUTHOR CONTRIBUTION

**Luis Romero Soto:** Data curation (equal); Formal analysis (equal); Investigation (equal); Methodology (equal); Writing‐original draft (lead). **Habib Thabet:** Investigation (supporting); Methodology (supporting); Visualization (equal). **Reuben Maghembe:** Data curation (equal); Formal analysis (equal); Methodology (equal); Validation (equal); Visualization (equal); Writing‐review & editing (equal). **Denise Gameiro:** Investigation (supporting); Methodology (supporting). **Doan Van‐Thuoc:** Investigation (equal); Methodology (supporting); Supervision (supporting); Validation (supporting); Writing‐review & editing (equal). **Tarek Dishisha:** Conceptualization (lead); Data curation (equal); Formal analysis (equal); Investigation (equal); Methodology (equal); Supervision (equal); Validation (equal); Visualization (equal); Writing‐review & editing (equal). **Rajni Hatti‐Kaul:** Conceptualization (supporting); Formal analysis (supporting); Funding acquisition (lead); Investigation (supporting); Project administration (lead); Resources (lead); Supervision (equal); Validation (supporting); Visualization (equal); Writing‐review & editing (lead).

## Data Availability

All data generated or analyzed during this study are included in the results section of this paper.

## APPENDIX A

**TABLE A1:** EPS biosynthetic pathways represented by gene ontology and annotation

Wizx/Wzy‐ dependent pathway					
Gene	NCBI Accession No.	Locus Tag	Uniprot Accession No.	Selected orthologs	Relevant functions
Glucans biosynthesis glucosyl‐transferase MdoH	NZ_CP014595.1	AYJ57_RS03310	A0A1B1PLL3	EAM_RS06910, A4W93_RS25685, BM397_RS00375	Transferase activity of glucosyl units and synthesis of glucan units
Glycosyl‐transferase family 4 protein	NZ_CP014595.1	AYJ57_RS13255	WP_083191252.1 (RefSeq)	BMF35_RS035	Transfer of glycosyl units between molecules and synthesis of oligosaccharides
Oligo‐saccharide flippase family protein (Wzx)	NZ_CP014595.1	AYJ57_RS13250	A0A1B1PRT3	CYCME_RS06905	Transmembrane transport of oligosaccharide molecules
	NZ_CP014598.1	AYJ57_RS22610	WP_083191479.1 (RefSeq)	M397_RS12935, BM397_RS12935	Polysaccharide biosynthesis and export
O‐antigen ligase family protein (Wzy)	NZ_CP014595.1	AYJ57_RS09970	A0A1B1PPY9	RPC_RS03340, B8987_RS08280, M397_RS10565	Polymerization, ligation of O‐antigen oligosaccharides to membrane lipid
Outer membrane polysaccharide export protein (OPX)	NZ_CP014595.1	AYJ57_RS07765	WP_083191183.1 (RefSeq)	SALMUC_RS10810, BM397_RS15370	Biosynthesis and export of polysaccharide

**ABC Transporter‐dependent pathway**					
**Gene**	**NCBI Accession No**	**Locus Tag**	**Uniprot ID**	**Orthologs**	**Relevant functions**
Exo‐polysaccharide biosynthesis protein	NZ_CP014598.1	AYJ57_RS22640	A0A1B1PXP9	SALMUC_RS20695, BM397_RS128	O‐acetylation, alginate biosynthesis, and protection
Polysaccharide export protein (Wza)	NZ_CP014595.1	AYJ57_RS07765	WP_083191183.1 (RefSeq)	SALMUC_RS10810, 397_RS15370	Poly‐saccharide export
Low molecular weight phosphor‐protein phosphatase (Wzb)	NZ_CP014596.1	AYJ57_RS15515	A0A1B1PTF7	ALMUC_RS04685, B8987_RS15445, BM397_RS24240, EAKF1_RS02225, NCTC86EC_RS03680	Wzc dephosphorylation, EPS colanic acid synthesis
Poly‐saccharide biosynthesis tyrosine autokinase (Wzc)	NZ_CP014598.1	AYJ57_RS22615	WP_083191480.1 (RefSeq)	EAKF1_RS02230, RPC_RS13440, NCTC86EC_RS09900	Tyrosine kinase activity, EPS biosynthesis, LPS biosynthesis

**The synthase pathway**					
Gene	NCBI Accession no	Locus tag	Uniprot accession no	Orthologs	Relevant function
Maltooligosyl‐trehalose synthase	NZ_CP014600.1	AYJ57_RS24360	A0A1B1PYG5	treY	Trehalose biosynthesis from maltodextrin
Tetra‐tricopeptide repeat protein	NZ_CP014595.1	AYJ57_RS06655	A0A1B1PN88	SALMUC_RS1611, BM397_RS16465	Polysaccharide assembly, ATP binding
Glycosyl‐transferase family 2 protein	NZ_CP014596.1	AYJ57_RS14955	WP_083191351.1 (RefSeq)	B8987_RS04440, BM397_RS14075	Lipase activity
